# Study on an Automatic Classification Method for Determining the Malignancy Grade of Glioma Pathological Sections Based on Hyperspectral Multi-Scale Spatial–Spectral Fusion Features

**DOI:** 10.3390/s24123803

**Published:** 2024-06-12

**Authors:** Jiaqi Chen, Jin Yang, Jinyu Wang, Zitong Zhao, Mingjia Wang, Ci Sun, Nan Song, Shulong Feng

**Affiliations:** 1 Changchun Institute of Optics, Fine Mechanics and Physics, Chinese Academy of Sciences, Changchun 130033, China; chenjiaqi19@mails.ucas.ac.cn (J.C.);; 2 University of Chinese Academy of Sciences, Beijing 130033, China

**Keywords:** hyperspectral, glioma, grade, feature extraction, neural network

## Abstract

This study describes a novel method for grading pathological sections of gliomas. Our own integrated hyperspectral imaging system was employed to characterize 270 bands of cancerous tissue samples from microarray slides of gliomas. These samples were then classified according to the guidelines developed by the World Health Organization, which define the subtypes and grades of diffuse gliomas. We explored a hyperspectral feature extraction model called SMLMER-ResNet using microscopic hyperspectral images of brain gliomas of different malignancy grades. The model combines the channel attention mechanism and multi-scale image features to automatically learn the pathological organization of gliomas and obtain hierarchical feature representations, effectively removing the interference of redundant information. It also completes multi-modal, multi-scale spatial–spectral feature extraction to improve the automatic classification of glioma subtypes. The proposed classification method demonstrated high average classification accuracy (>97.3%) and a Kappa coefficient (0.954), indicating its effectiveness in improving the automatic classification of hyperspectral gliomas. The method is readily applicable in a wide range of clinical settings, offering valuable assistance in alleviating the workload of clinical pathologists. Furthermore, the study contributes to the development of more personalized and refined treatment plans, as well as subsequent follow-up and treatment adjustment, by providing physicians with insights into the underlying pathological organization of gliomas.

## 1. Introduction

Diffuse gliomas are believed to derive from glial stem cells and are the most common primary brain tumors. Based on their histological characteristics, gliomas are classified as astrocytes, oligodendrocytes, or ependymal tumors, and their malignancy is designated as I to IV by the World Health Organization (WHO) [1]. Recent progress in the genetic analysis of brain tumors has demonstrated that they differ greatly with respect to morphology, location, genetic characteristics, and response to treatment. Histopathology is the gold standard for glioma grading, but the histological classification and grading of diffuse glioma present many challenges for pathologists, and reliance on traditional histology to grade gliomas carries some disadvantages. First, these tumors differ greatly between observers and within observers [2]. Because the diagnostic criteria are not accurate, this variability leads to poor consistency and repeatability of tumor grading. Moreover, diffuse gliomas often show obvious phenotypic heterogeneity and present spatial differences in cell phenotype and anaplastic degree, which leads to uneven distribution of mitotic images. This tendency leads to a high rate of variation among observers in the diagnosis of diffuse gliomas, including oligodendrocytoma (ODG) [3]. Because of these factors, it is not possible to accurately identify the biological specificity of tumors.

In recent years, many studies have submitted pathological specimens of brain gliomas to computer algorithms for machine-aided diagnosis, such as deep convolutional neural networks. This technology has been used to detect tumor cells, classify tumor subtypes, and diagnose diseases [4,5]. The motivation for adopting this technology is to provide rapid, reproducible, and quantitative diagnosis [6]. Zhou et al. established a convenient and non-invasive method for preoperative grading of glioma [7]; Their method, which relies on MRI proteomics technology combined with enhanced T1WI, substantially improved the diagnostic accuracy. Zhang et al. used machine learning to explore the potential of MRI imaging features for distinguishing anaplastic oligodendroglioma (AO) from atypical low-grade oligodendroglioma, and established a prediction model based on T1C and FLAIR image features [8]; Gao et al. introduced a non-invasive method for preoperative prediction of glioma grade and expression levels of various pathological biomarkers, achieving good prediction accuracy and stability [9]. A large number of training datasets is often needed to achieve effective classification recognition, and detection efficiency is relatively low. The new (2016) WHO classification criteria for central nervous system tumors marked the end of traditional diagnostic methods solely based on histological standards and molecular biomarkers that combined morphological and molecular features from diffuse glioma [10]. Therefore, there is an urgent need for an objective, stable, and reproducible method to accurately grade diffuse gliomas that faithfully reflect the essential attributes of tumors, thus effectively guiding clinical surgery and prognosis treatment.

Micro-hyperspectral images carry important information about biological tissues and can be used to analyze their biochemical properties. Previous research has achieved automated detection of various cancers, including visualization enhancement of blood vessels, by combining hyperspectral imaging with deep learning [11,12]. This research has made it possible to identify intestinal ischemia [13], measure the oxygen saturation of the retina [14], estimate the human cholesterol level [15], and perform cancer testing [16,17,18,19]. Hyperspectral images carry many advantages compared with traditional medical images. Xuehu Wang [20] proposed an automatic detection method for predicting hyperspectral characteristics based on radiological characteristics of human computed tomography (CT) for the non-invasive detection of early liver tumors. Through image enhancement and XGBoost model construction, the mapping relationship between CT characteristics and corresponding hyperspectral imaging (HSI) data is learned, and good classification and recognition results are achieved. Wang [21] proposed a feature fusion network of RGB and hyperspectral dual-mode images (DuFF-Net), in which features were mined using SE-based attention modules and Pearson correlation. The results showed that DuFF-Net improved the screening accuracy of two morphologically similar gastric precancerous tissues, reaching accuracy values up to 96.15%. Jian et al. [22] introduced a spectral space transfer convolutional neural network (SST-CNN) to address the issue of limited sample size of medical hyperspectral data. This method achieved classification accuracy values of 95.46% for gastric cancer and 95.89% for thyroid cancer.

In light of the above results, we designed a multi-modal and multi-scale hyperspectral image feature extraction model (SMLMER-ResNet) based on microscopic hyperspectral images of brain gliomas. In our model, the channel attention mechanism (SE module) is introduced before the traditional residual connection module, to explore interactions among spectral channels in the three-dimensional data cube of hyperspectral images of brain glioma from pathological slices. This design was chosen to realize the fusion of features between spectral channels and, therefore, improve the feature extraction ability of the network, while effectively reducing redundant information. Building on this architecture, we introduced the parallel multi-scale image feature method to further extract features of hyperspectral images from the three-dimensional data cube. We also improved the convolutional layer in the residual connection by introducing multi-branch modules with different image sizes. This design characteristic not only achieves deep data feature mining but also realizes a lightweight model. Our model delivered accurate and efficient identification of diffuse glioma from pathological sections, in conformity with the classification criteria introduced by the WHO in 2016.

## 2. Materials and Methods

### 2.1. Dataset Structure

**Figure 1:**
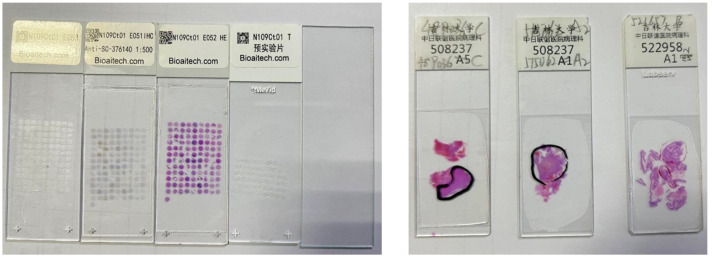
Diagram of glioma pathological chip array.

**Figure 2:**
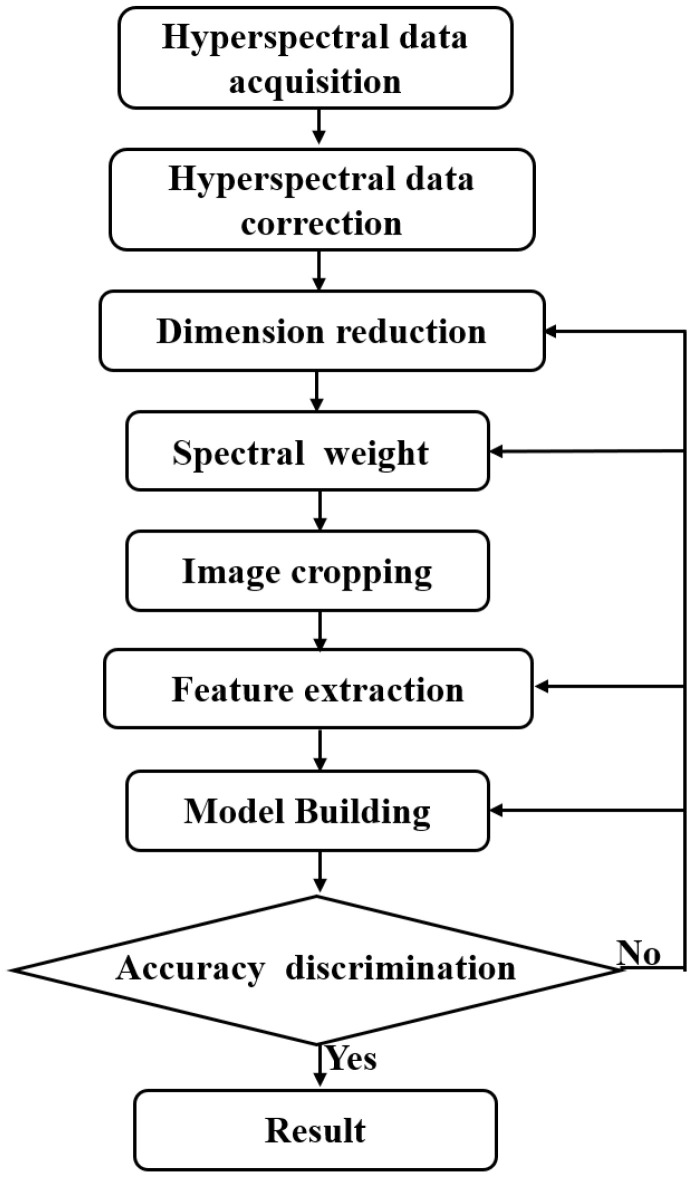
Brain glioma processing flow.

All sections were digitized and magnified using an Olybas 40× objective lens. They were manually annotated by a pathologist with professional certification. We used cancer and normal regions as marked on digital histological images as ground truth for data selection and classification. For each slide, we selected at least three ROIs within the cancer region, which were marked by pathologists specializing in relevant research. In Figure 1, we collaborated with staff at a tertiary hospital in Changchun, to complete formalin fixation 1, HE staining, IHC staining, and sample labeling of brain gliomas. We had access to 109 cases of brain gliomas spanning different sexes and ages (14 cases of grade 1, 64 cases of grade 2, 46 cases of grade 3, and 72 cases of grade 4), together with 22 normal brain tissues. The processing is shown in Figure 2.

### 2.2. Micro-Hyperspectral Data Collection

**Figure 3:**
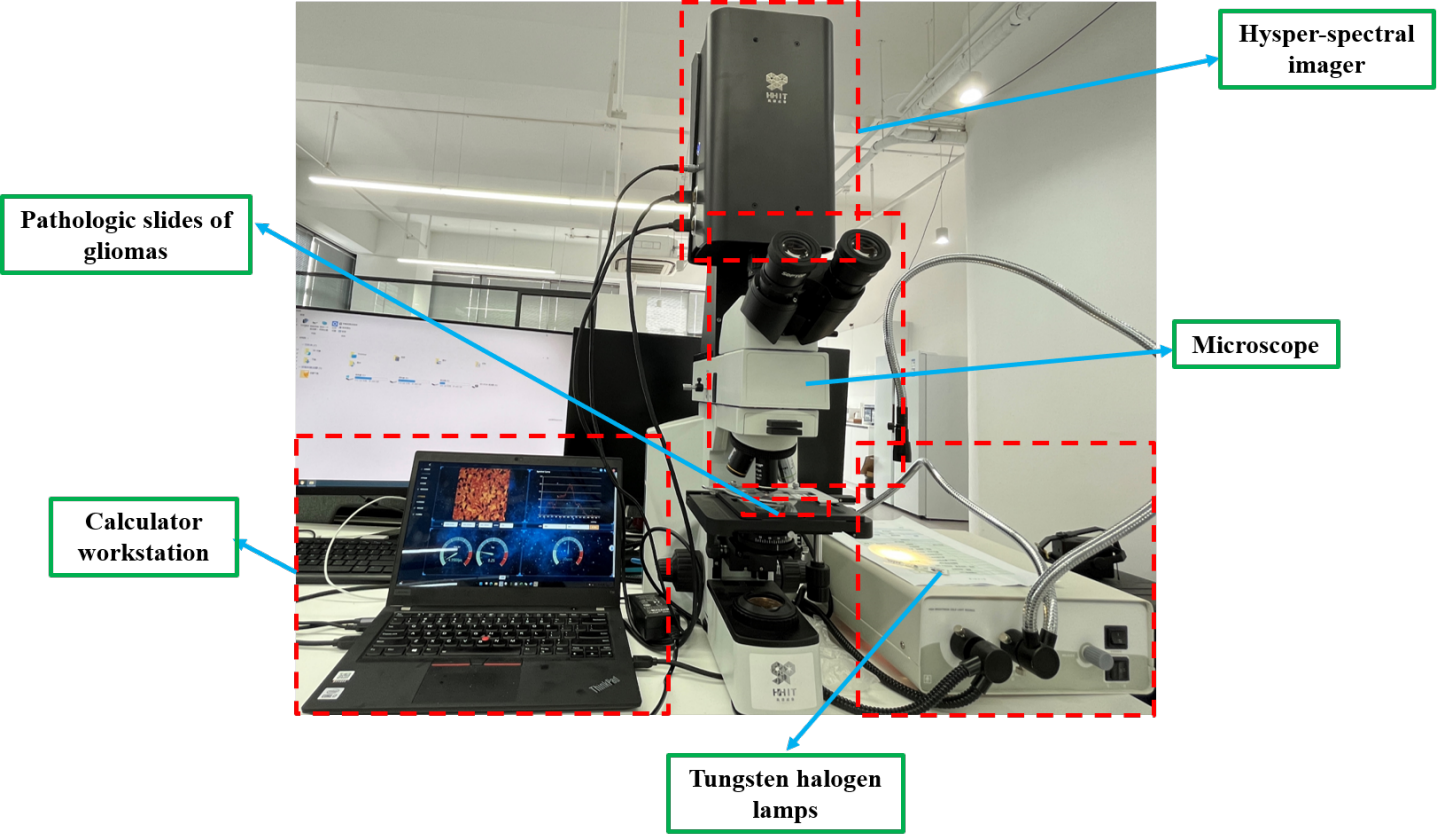
Schematic diagram of a microscopic hyperspectral acquisition system.

**Figure 4:**
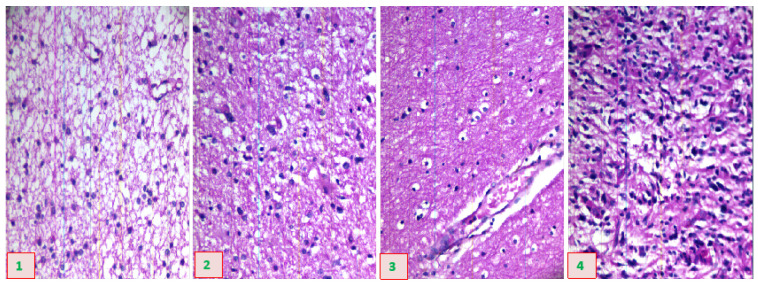
Diagram of hyperspectral data images from pathological sections of brain gliomas of different grades.

We obtained HSIs from the slides of brain glioma using the micro-hyperspectral imaging system integrated by Hangzhou Hyperspectral Imaging Technology Co., Ltd. in Hangzhou, China. The hyperspectral imaging module was independently developed by the research group of Changchun Institute of Optics, Fine Mechanics, and Physics (CIOMP), Chinese Academy of Sciences, as shown in Figure 3. The acquisition system consists of an imaging system, an illumination system, and a computer. In the spectral imaging system, the objective lens used is the Olympus PLN series achromatic objective lens with a magnification of 40×, and the detector is a CMOS two-dimensional area array camera manufactured by imperx, Inc. of Boca Raton, FL, USA. The spectral range of the system covers 400–1000 nm, the number of spectral channels is 270, and the spectral resolution is >2.5 nm. After push-scan imaging, the system can capture a three-dimensional data cube of the specimen. The lighting system consists of a cold light source of optical fiber halogen tungsten lamp with a power of 150 W, and the spectral range of the continuous spectrum light source is 400–2200 nm. Figure 4 provides a schematic diagram of hyperspectral data images from glioma pathological sections of different grades, collected using the system just described. The images show a grade 1 pathology sample of a female astrocytoma aged 43 years, a grade 2 pathology sample of a male astrocytoma aged 43 years, a grade 3 pathology sample of a female astrocytoma aged 40 years, and a grade 4 pathology sample of a female glioblastoma aged 46 years.

### 2.3. Spectral Data Preprocessing

**Figure 5:**
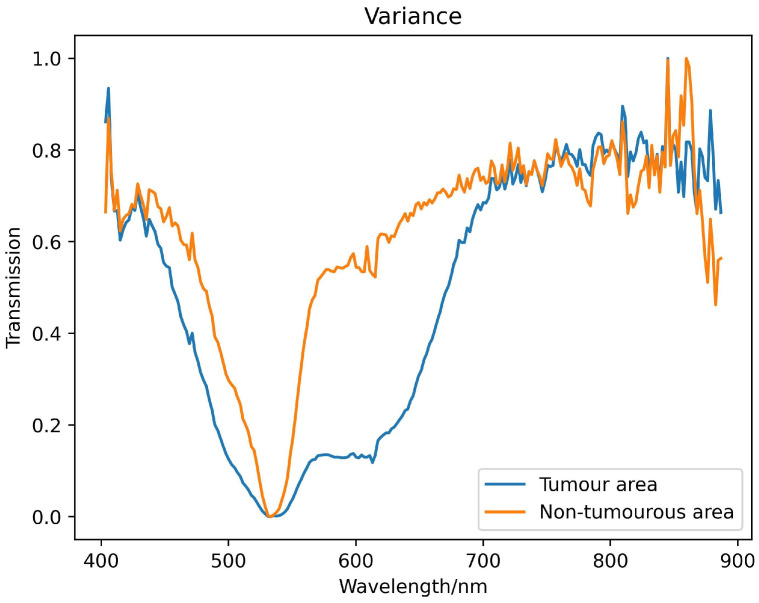
Example spectral reflectance curve obtained from a pathological section of brain glioma.

We used hyperspectral images of brain glioma tissue array chip to create the database used in this study. In order to reduce system noise, the radiation intensity difference of the pixel response, and the influence of light source instability, while at the same time preserving spectrum information as much as possible, we correct system transmittance based on Lambert-Beer law [23] as detailed in Formula (1), where *T* is the transmittance data after spectral correction, representing both the original sample data and the blank slide data under the same experimental conditions. $I_{O}$ represents the radiant intensity transmitted by the blank slide. $I_{W}$ represents the radiant intensity transmitted by the glioma slides, and $I_{S}$ represents dark noise intensity. Finally, we implemented normalization and SG filtering. This process ensures data consistency and repeatability of algorithm results. The spectral transmittance curve obtained after preprocessing is shown in Figure 5.

(1) $$T=\left(I_{W}-I_{S}\right)/\left(I_{O}-I_{S}\right)$$

### 2.4. Experimental Setup

Our hardware consisted of a GPU (NVIDIA Geforce RTX208Ti) manufactured by NVIDIA Corporation, Santa Clara, CA, USA. We used Windows 11 as the operating system, python 3.7 as the programming language, and pytorch as the deep-learning framework. We adopted the following parameters: the parametric ReLU activation function, a batch size of 32, epochs equal to 100, and the ratio of the test set, validation set, and training set being 4:1:5. The study utilized Adam as the optimizer. Additionally, due to the intricacy of the medical sample production process and the requirement for professional doctors to annotate the images, there were limited samples available for training. Therefore, regularization was implemented to decrease the network’s complexity, prevent overfitting, and enhance the model’s generalization ability. To improve classification accuracy and avoid overfitting, we utilize a layer-specific regularization and normalization classification mechanism. This mechanism includes a fully connected layer, a sigmoid activation function, and an L2 regularization function. The exact mathematical expression is shown in Formula (2).

(2) $$y=\sigma \left(\omega_{2}\sigma \left(\omega_{1}\left(y_{s}\right)+\lambda {∥\omega_{1}∥}_{F}^{2}\right)\right)$$

where $\omega_{2}$ and $\omega_{1}$ denote the kernels of the two fully connected layers, respectively; λ is the L2 regularization parameter of the fused feature weights; σ denotes the sigmoid activation function; and $y_{s}\;$ and *y* denote the inputs and outputs of the classifier, respectively. The mathematical expression for the cross-entropy function is shown in Formula (3).

(3) $$L=\frac{1}{T}\sum_{i=1}^{T}\sum_{j=1}^{K}t_{j}^{i}\log\left(\frac{e^{w_{k}y^{y}+b_{k}}}{\sum_{m=1}^{K}e^{w_{m}y^{j}+b_{m}}}\right)$$

where *w* denotes the weight and *b* denotes the bias of the current layer, respectively, $y^{i}$ denotes the output of the *i*th training sample, and *T* denotes the number of training samples. This provides the necessary guarantee for later modeling of high accuracy in delineating the malignant grade of gliomas.

We evaluated classification performance using overall accuracy (OA), average accuracy (AA), and the Kappa coefficient. OA represents the ratio between the total number of correctly classified samples and all tested samples. AA represents the average classification accuracy across all categories. Kappa coefficient is used to evaluate classification consistency across all categories. To eliminate the influence of random factors, all experimental results in this study are obtained from an average of 10 experiments.

### 2.5. Data Dimensionality Reduction

The redundancy associated with hyperspectral data may lead to a decrease in the classification efficiency of malignant grades. In this study, we used the simulated annealing algorithm and principal component analysis to select the appropriate wavelength. In this procedure, the annealing algorithm is simulated by defining an appropriate energy function, which is an objective function. The new wavelength subset can be accepted or rejected according to the probability of energy change, and the most suitable wavelength subset can be gradually optimized to achieve the purpose of wavelength dimensionality reduction. The successive projections algorithm (SPA) [24] utilizes projection analysis of vectors by projecting wavelengths onto other wavelengths, comparing the magnitude of the projection vectors, taking the wavelength with the largest projection vector as the wavelength to be selected, and then selecting the final feature wavelength based on the correction model. SPA selects the combination of variables that contains the least amount of redundancy information and the least amount of covariance. We used several different machine learning methods to control variables, including support vector machines (SVM) [25], decision tree classifier (RT) [26], random forest (RFR) [27], and selected the data dimensionality reduction method that was most suitable for this study. Relevant results are shown in Table 1. The simulated annealing algorithm is clearly superior to the SPA algorithm for different classification models.

### 2.6. Identify Model

**Figure 6:**
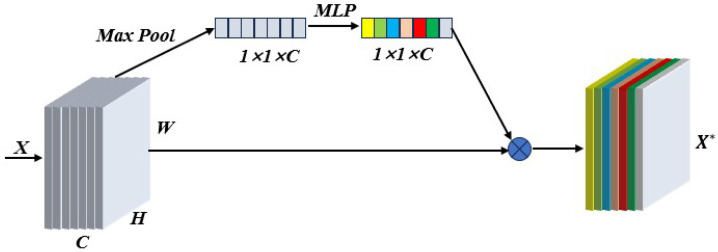
Procedure adopted to implement the attention mechanism in different spectral channels.

Based on the results from the previous section, we performed a partial decision tree (DT), SVM classification, RFR, one-dimensional convolutional neural network (CNN) and MSE-ResNet [28]. We used the improved algorithm to establish the classification model for grading pathological sections of glioma with different malignant grades, which we term SMLMER-ResNet. We used support vector classification (SVC) to establish models for classifying pathological sections of gliomas with different malignant grades. SVC is a typical nonlinear classification method, which maps low-dimensional data to high-dimensional space and realizes the classification of samples by finding the optimal hyperplane. In SVC, the appropriate choice of the penalty coefficient *C* and kernel function *G* ensures strong generalization ability [29]. We used a genetic algorithm to optimize the penalty coefficient *C* and kernel function parameter *G*. A decision tree (decision tree) is a predictive analytics model expressed in the form of a tree structure (including binary and multinomial trees). Each non-leaf node represents a test on a feature attribute, each branch represents the output of this feature attribute on a certain value domain, and each leaf node stores a category. The random forest algorithm consists of several decision trees, with no correlation between decision trees in the forest. The final output of the model is determined by all decision trees, which carry strong anti-interference ability and anti-over-fitting abilities. Jian [30] used spectral information from microscopic hyperspectral images to classify gastric cancer tissue via 1D-CNN. The network model included one input layer, two convolution layers, two pooling layers, one fully connected layer, and one output layer. Chongxuan et al. [31] relied on hyperspectral images of gastric juice to classify Helicobacter pylori infection and used a ResNet model to learn from two-dimensional data, achieving 91.48% classification accuracy. We improved ResNet by introducing a SE module after the pretreatment process, such as SAA, and used it to learn the relationship between channels by assigning corresponding weights to each channel, to realize the effective fusion of features between channels. Figure 6 shows the adopted implementation process, which can effectively reduce redundant information and improve the feature extraction ability of the network.

**Figure 7:**
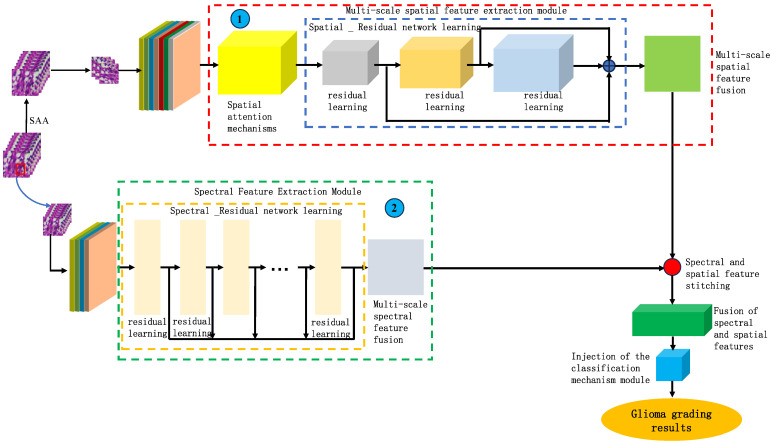
The overall framework of the designed model.

**Figure 8:**
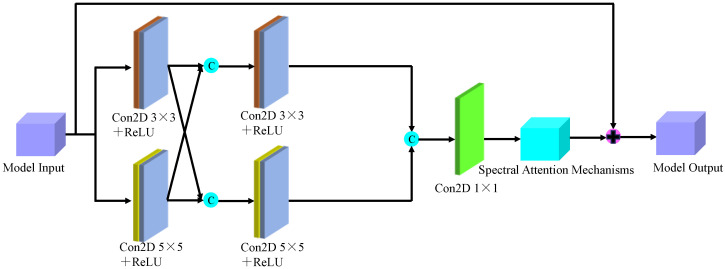
The multi-scale spectral feature extraction framework.

Based on the findings of this study, we proposed a model for identifying the malignancy level of glioma pathological tissues by integrating the basic framework of SENet and ResNet residual network models. This model, named SMLMER-ResNet (neural network modeling of multimodal multiscale hierarchical spectral–spatial fusion features), combines spectral–spatial multiscale features. Figure 7 shows the overall framework of the designed model, The overall network architecture comprises four main modules: spectral feature extraction, spatial feature extraction, higher-order spatial–spectral fusion features, and a classifier module. ➀ represents the spectral feature extraction module, which introduces residual networks and spectral attention mechanisms. ➁ represents the spatial feature extraction module, which incorporates residual networks and spatial attention mechanisms. Figure 8 depicts the multi-scale spectral feature extraction framework. In this study, we inputted an image block of size 7 × 7 × 200 into the spectral feature extraction module. Initially, an initialization process was conducted, consisting of a convolutional layer with 64 filters of size 3 × 3, a BN layer, and a ReLU activation function. Following the initialization module, the size of the original 3D data cube was changed to 7 × 7 × 64. Subsequently, the downscaled transformed data cube was further inputted into the multiscale spectral feature extraction module to capture multiscale spectral features and eliminate redundant information interference. By integrating six combined SE modules with the residual learning network, we achieved multi-scale spectral feature extraction, resulting in six local spectral features of size 7 × 7 × 64. Finally, global spectral features were obtained through spectral feature fusion. The multiscale spectral feature fusion module employed a combined processing strategy involving cascade operation and bottleneck layer, with the bottleneck layer consisting of 64 2D convolution kernels of size 1 × 1. Consequently, a multiscale spectral feature matrix of dimensions 7 × 7 × 64 was obtained on the spectral feature extraction branch.

**Figure 9:**
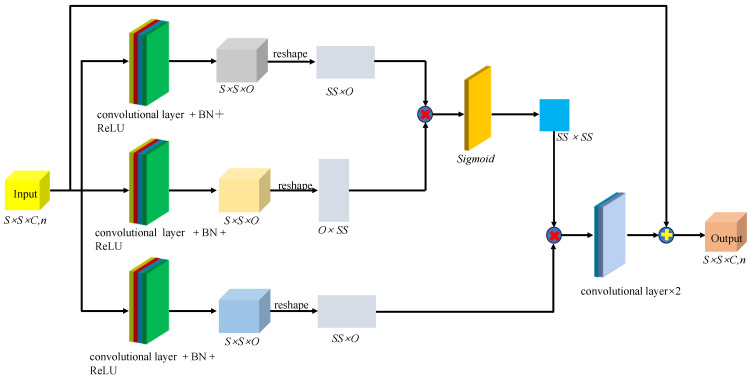
The multi-scale hierarchical spatial feature extraction module.

Figure 9 shows the multi-scale hierarchical spatial feature extraction module. This study adds spatial attention mechanisms to the module. The spatial-level attention mechanism aims to identify which spatial pixel regions are worth focusing on and reassign weights to the spatial context information areas. $X_{k}\in R^{S\times S\times C}$ represents the input data for the spatial-level attention mechanism, where S×S and $C_{1}$ denote the spatial dimensions and the number of spectral bands, respectively. First, to reduce computational complexity and the number of spectral bands, a 3D convolutional layer with a size of 1×1×C×O is used to transform the input data, $X_{k}\in R^{S\times S\times C}$ in a top-down order, resulting in $f\left(X_{k}\right)\in R^{S\times S\times O}$, $g\left(X_{k}\right)\in R^{S\times S\times O}$, and $h\left(X_{k}\right)\in R^{S\times S\times O}$. The mathematical expression of $f\left(X_{k}\right)$ can be written as follows:

(4) $$f\left(X_{k}\right)=\delta \left(\omega_{f}*X_{k}+b_{f}\right)$$

Here, $\omega_{f}$ and $b_{f}$ represent the weights and biases of the 3D convolutional layer, respectively. The mathematical expressions for $g\left(X_{k}\right)$ and $h\left(X_{k}\right)$ are similar to those of $f\left(X_{k}\right)$. Next, the feature map $f\left(X_{k}\right)\in R^{S\times S\times O}$ is reshaped into $f\left(X_{k}\right)\in R^{SS\times 0}$. By calculating the product of $f\left(X_{k}\right)$ and $g{\left(X_{k}\right)}^{T}$, the interdependencies between different hyperspectral pixels are obtained, and its mathematical expression can be written as follows:

(5) $$R=f\left(X_{k}\right)g{\left(X_{k}\right)}^{T}$$

Subsequently, a softmax function is used to normalize *R*, which can be written as follows:

(6) $$\hat{R}\left(i,j\right)=\frac{e^{R(i,j)}}{\sum_{j=1}^{SS}e^{R(i,j)}}$$

Further, by calculating the product of the normalized $\hat{R}$ and $h\left(X_{k}\right)$, the attention feature map Att is generated. Finally, using two 3D convolutional layers with sizes 1×1×C×O and 1×1×n×n, $Att\in R^{S\times S\times O}$ is transformed into $Att^{\prime }\in R^{S\times S\times C}$. Additionally, to enable the reuse of shallow features and promote information flow, skip connections are introduced into the spatial-level attention mechanism.

Additionally, this study incorporates a hierarchical spatial feature fusion module, recognizing that spatial features captured at different stages have varying importance. Shallow features have a smaller receptive field and can only extract local features, but they possess high resolution and rich detail information. In contrast, deep features have a lower resolution but contain more abstract high-level semantic information. The characteristics of shallow and deep features are complementary. Inspired by this, we designed a hierarchical spatial feature fusion module to aggregate spatial features from different stages, thereby obtaining multi-level spatial features.

The hierarchical spatial feature fusion module consists of three parts: the low-level residual module, the mid-level residual module, and the high-level residual module. It can capture both detailed shallow spatial features and abstract deep spatial features. Each residual module is composed of two 3D convolutional layers, one BN layer, and a PReLU activation function. The convolutional layers have 16 filters of size 3 × 3 × 1. The BN layer and PReLU activation function are used to enhance the network’s nonlinear discrimination ability. Additionally, to accelerate model convergence and avoid overfitting, skip connections are introduced in each module. $I^{i}$ and $O^{i}$, represent the input and output data of the residual modules, respectively. i∈[0,1,2], and *i* is the index of the residual module The basic principle of the hierarchical spatial feature fusion module can be written as follows:

(7) $$x^{0}=\omega_{1}^{0}\left(\delta \left(\omega_{0}^{0}*I^{0}+b_{0}^{0}\right)\right)+b_{1}^{0}$$

(8) $$O^{0}=I^{0}+x^{0}$$

(9) $$x^{1}=\omega_{1}^{1}\left(\delta \left(\omega_{0}^{1}*O^{0}+b_{0}^{1}\right)\right)+b_{1}^{1}$$

(10) $$O^{1}=O^{0}+x^{1}$$

(11) $$x^{2}=\omega_{1}^{2}\left(\delta \left(\omega_{0}^{2}*O^{1}+b_{0}^{2}\right)\right)+b_{1}^{2}$$

(12) $$O^{2}=O^{1}+x^{2}$$

(13) $$O=O^{0}+O^{1}+O^{2}$$

Here, $x^{i}$ represents the intermediate output of the *i* residual module; ω and *b* denote the weights and biases of the convolutional layers, respectively, with their superscripts indicating the index of the residual module and subscripts indicating the index of the current convolutional layer. δ represents the PReLU activation function. The final output is achieved through additional operations to compensate for information loss during network computations.

In this module, the original micro-hyperspectral data of glioma pathology were initially downscaled using SAA, and image blocks of size 27 × 27 × 30 were inputted. Similar to the spectral feature extraction process, an initialization process was performed, comprising a 3D convolutional layer, a BN layer, and an activation function containing 16 filters of size 5 × 5 × 30 and 16 filters of size 3 × 3 × 1, with ReLU as the activation function. After the initialization module, the original input data were transformed into a four-dimensional vector, and the size of the dimensions 23 × 23 × 1 × 16 was calculated. The transformed data were then inputted into the spatial feature extraction module with a spatial attention mechanism to achieve multilevel spatial feature extraction. The multi-scale spatial feature extraction module included a spatial attention mechanism and a multi-stage spatial feature fusion module. After multiscale spatial feature extraction, the spatial feature size was changed to 23 × 23 × 16 × 16, followed by spatial feature fusion. To be spliced with spectral features from another branch, the spatial features were transformed into 7 × 7 × 8 of multilevel spatial features.

**Figure 10:**
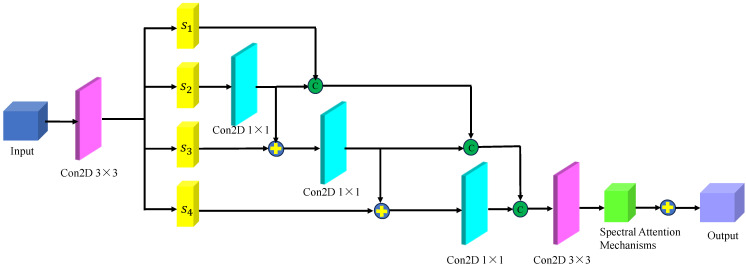
The multi-scale spectral feature extraction framework.

Figure 10 shows the spatial–spectral fusion feature extraction module. This module effectively captured the close connection between spatial–spectral features, integrating multiscale spectral features, multilevel spatial features, and higher-order fusion features for further refinement and extraction. After processing, more comprehensive and integrated multiscale features were obtained for analyzing and identifying the malignancy level of glioma pathology sections. Specifically, the multi-scale spectral features and multi-level spatial features are aggregated using a cascade operation, and the resulting spectral–spatial joint features serve as the input for this module. Then, after a 1 × 1 2D convolutional layer, the spectral–spatial joint features are evenly divided into four subsets as input data for different branches, denoted as $s_{1}$, $s_{2}$, $s_{3}$, $s_{4}$. Each subset contains one-fourth of the original input data channels. Next, $s_{2}$ is passed to the convolutional layer on the second branch to generate the first high-level semantic feature subset ${\hat{s}}_{2}$, where the convolutional layer has 18 filters of size 3 × 3. Subsequently, the input data for the convolutional layer on the third branch is obtained by adding ${\hat{s}}_{2}$ and $s_{3}$, and the input data are processed through convolution to generate the second high-level semantic feature subset ${\hat{s}}_{3}$. Similarly, we add ${\hat{s}}_{3}$ and $s_{4}$ and pass the result to the convolutional layer on the fourth branch to generate the third high-level semantic feature subset ${\hat{s}}_{4}$. The basic principle of this module can be written as follows:

(14) $${\hat{s}}_{i}=\left\{\begin{array}{cc} s_{i} & i=1\\ k_{i}\left(s_{i}\right) & i=2\\ k_{i}\left({\hat{s}}_{i-1}+s_{i}\right) & 2<i\leq 4 \end{array}\right.$$

where $k_{i}$ denotes the convolution operation. Finally, we use a cascade operation to aggregate $s_{1}$, ${\hat{s}}_{2}$, ${\hat{s}}_{3}$, ${\hat{s}}_{4}$, and pass them to a 1 × 1 convolutional layer. Finally, to prevent overfitting of the model after deep-level feature extraction, we applied layer-specific regularization and normalization to the classifier, resulting in adaptive adjustment of the weights of the fused features. Given that the glioma pathology slice dataset contained 5 categories, the model outputs a one-dimensional vector of dimension 5.

## 3. Results

### 3.1. Model Parameter Analysis

#### 3.1.1. Image Block Size Analysis

**Figure 11:**
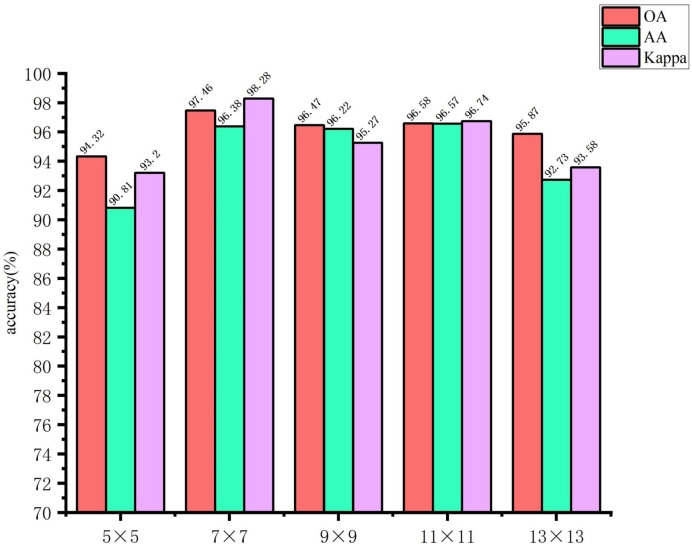
The input image block sizes of the spectral feature extraction module.

**Figure 12:**
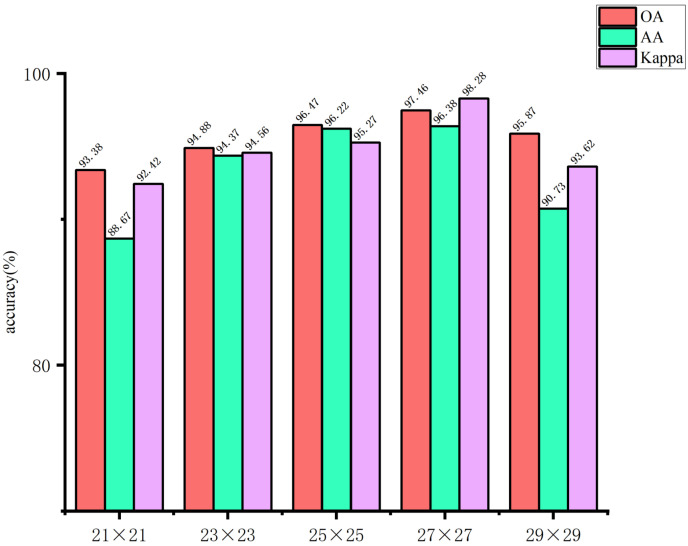
The input image block sizes of the spatial feature extraction module.

The use of larger image blocks can facilitate the capture of more complex features by the network. However, if the image block is too large, it may introduce irrelevant information, which could affect the extraction of spatial features for small targets, thereby hindering the network’s ability to learn features useful for classification. Conversely, if the image block size is too small, it may not be possible to extract sufficient spatial information, which could result in a decline in the overall classification accuracy of the model. Accordingly, in this study, the input image block sizes of the spectral feature extraction module were set to 5 × 5, 7 × 7, 9 × 9, 11 × 11, and 13 × 13, respectively. The input image block sizes of the spatial feature extraction module are set as 21 × 21, 23 × 23, 25 × 25, 27 × 27, and 29 × 29. The impact of the spatial dimensions of the model input image blocks on the classification outcomes of the glioma pathology sample dataset is illustrated in Figure 11 and Figure 12. These figures demonstrate that when the spectral feature extraction module inputs an image block with a size of 7 × 7, while the spatial feature extraction module inputs an image block with a size of 27 × 27, the SMLMER-ResNet model proposed in this study exhibits superior performance metrics on the glioma pathology sample dataset. The input image block size for the spatial feature extraction module is 27 × 27, while the input image block size for the spectral feature extraction module is 7 × 7. The SMLMER-ResNet model proposed in this study exhibits superior performance metrics on the glioma pathology sample dataset. Consequently, the optimal input image block sizes for the SMLMER-ResNet network model were determined to be 7 × 7 (the input image block size for the spectral feature extraction module) and 27 × 27 (the input image block size for the spatial feature extraction module), respectively. This was done by considering the classification accuracy and the amount of network computation.

#### 3.1.2. Model Learning Rate Analysis

**Figure 13:**
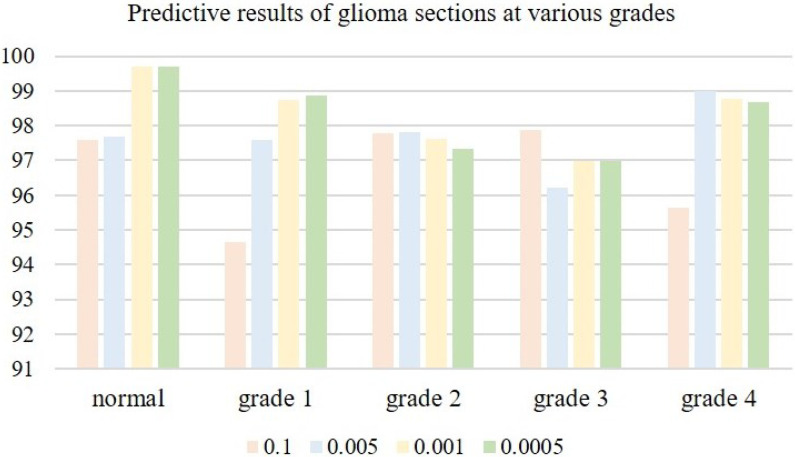
Predictive results of glioma sections at various grades.

The choice of an appropriate learning rate can help the network model deliver better training results. When the learning rate is very small, the convergence speed is slow, and overfitting can easily occur; when the learning rate is high, the gradient may oscillate back and forth around the minimum value, and may even fail to converge. We tested four learning rates on microscopic hyperspectral data from pathological sections of brain glioma: 0.01, 0.005, 0.001, and 0.0005. Classification results associated with different learning rates are shown in Table 2. As the learning rate is decreased, classification accuracy gradually improves. When the learning rate is too small, the convergence speed of the model slows down, and classification accuracy decreases. When the learning rate is 0.001, the model reaches its peak classification accuracy in Figure, as is shown in Figure 13. We, therefore, settled on a learning rate of 0.001.

### 3.2. Ablation Experiment

The SMLMER-ResNet framework is comprised of four modules: a spectral feature extraction module, a spatial feature extraction module, a multiscale spatial–spectral feature fusion module, and a classifier module. In order to comprehensively assess the efficacy of these modules, we have designed model ablation experiments based on the micro-hyperspectral dataset of glioma pathology slices. We have initiated the validation process by examining each module in isolation with respect to the following criteria.

#### 3.2.1. Effectiveness of Classification Mechanisms

In order to ascertain the efficacy of the proposed classifier, a series of ablation experiments were devised in this study to test the following distinct classifier configurations: The following configurations were tested: (1) Fully connected layer with ReLU, FR; (2) fully connected layer with sigmoid, FS; (3) fully-connected layer, ReLU + L2 regularization, FRL2; (4) the classifier designed in this study, which combines the fully-connected layer, the sigmoid activation function, and the L2 regularization function, FSL2. Table 3 illustrates that the classifier method proposed in this paper outperforms traditional classifiers in classifying the malignant level of glioma pathology tissue. Furthermore, the model evaluation indexes are superior to those of other mechanisms.

#### 3.2.2. Effectiveness of Attention Mechanisms

In order to demonstrate the efficacy of the attention mechanism (spectral and spatial) of the model under investigation, an ablation experiment is conducted in this section. The ablation experiment incorporates the classification model into the aforementioned modules, namely the module without attention mechanism, the module with spectral attention mechanism, the module with spatial level attention mechanism, and the multimodal multiscale attention mechanism, as designed in this study. Table 4 illustrates that the model without an attention mechanism exhibits inferior performance in terms of OA, AA, and Kappa compared to the model with an attention mechanism. This evidence supports the necessity of introducing an attention mechanism. Additionally, the model with the introduction of the spectral attention mechanism demonstrated comparable performance to the model with the introduction of the spatial attention mechanism. However, the evaluation indexes are substantially worse than those of the model with the introduction of the multimodal and multi-scale attention mechanism designed in this paper. This is sufficient to prove that the method proposed in this study is more effective than the model with the introduction of a spatial-level attention mechanism.

### 3.3. Comparison between the Improved Model and the Traditional Model

**Figure 14:**
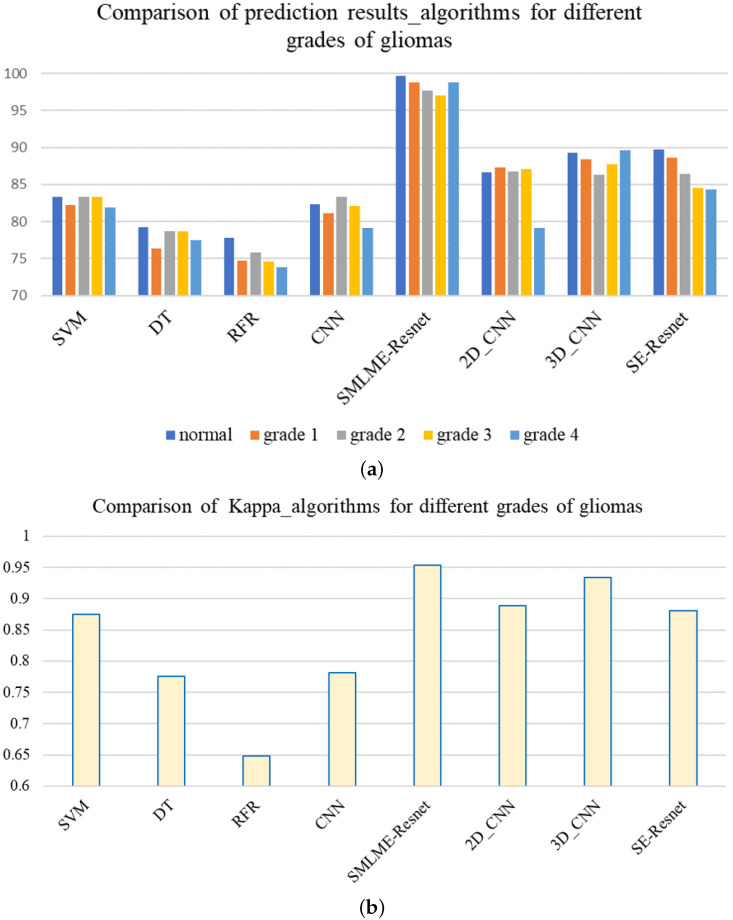
Feature extraction process of hyperspectral images of pathological sections of gliomas of different grades. (**a**) OA%, (**b**) Kappa.

In order to verify the improvement in model performance, we compared the traditional SE-ResNet algorithm with the SMLMER-ResNet algorithm proposed in this study for its ability to classify pathological sections of brain gliomas at different levels. We used the same dataset and preprocessing steps for the two models. The classification results are shown in Table 5, and recognition results for different algorithms are shown in Figure 14. Overall accuracy (OA), average accuracy (AA), and Kappa coefficient of SMLMER-ResNet are superior to those produced by SE-ResNet and are much higher than those produced by other machine learning algorithms. These results demonstrate that the improvements introduced by our study can extract spatial–spectral features from brain gliomas pathological slices of different levels more effectively. SMLMER-ResNet has slightly more tunable parameters (126,540) than SE-ResNet (123,780), but accuracy has been greatly improved, as shown in Table 5, demonstrating that the SMLMER-ResNet method proposed in this study can retain the lightweight nature of the original model while achieving better accuracy in identifying malignant grades in Figure 14.

## 4. Discussion

In recent years, machine learning methods, especially deep learning algorithms, have become an emerging technology to assist automated diagnosis in pathology. It can increase the speed of diagnosis and reduce inter-pathology variability [32,33,34,35,36]. Previous studies have mainly used larger RGB image blocks and low magnification to include sufficient histologic features. Relatively complex network architectures are generally required to effectively classify these image blocks. HSI technology can capture small spectral differences caused by molecular changes, providing more useful information than traditional three-channel RGB tissue color information. As a result, HSI technology can benefit many different microscopy applications, such as cancer detection in tissue sections.

This study focuses on the identification of malignant grade from pathological sections of glioma using microscopic hyperspectral images. We combine hyperspectral images with neural network algorithm properties at a deep level to accomplish the identification and classification of pathological sections of different subtypes of gliomas. We introduce a multimodal and multiscale hyperspectral image feature extraction model, SMLMER-ResNet, which integrates a channel attention mechanism. This model comprises a multiscale spectral feature extraction module, a hierarchical spatial feature extraction module, and a null-spectral fusion feature extraction module. These modules are designed for the classification of microscopic hyperspectral images of glioma pathology slices. The SMLMER-ResNet module first assigns weights to the features of each channel to learn feature correlations between different channels, thus realizing the fusion of features across channels. The original convolution layer is replaced by a maximum pooling layer and by an up-sampling layer to extract salient features of the image, and features at different scales in the channel dimension are extracted by a parallel feature fusion method. The 1 × 1 convolutional layer is added at the level of the jump connection to compensate for the local information lost within the pooling layer. Concurrently, we extracted both shallow and deep semantic features of glioma pathology tissue sections, thereby laying the foundation for improving model accuracy. We first selected the best data dimensionality reduction method (SAA) through machine learning. In that process, we found that the accuracy of subtype identification in pathological sections of gliomas in the brain was no more than 90% when different dimensionality reduction methods were combined with classification modeling methods, and the SAA-SVM, SAA-DT, and SAA-RFR models outperformed the SPA-SVM, SPA-DT, and SPA-RFR models in terms of both accuracy and Kappa. We designed our experiments to identify the optimal parameters of the SMLMER-ResNet model. We set the image block size to 7 × 7 and 27 × 27. We set the learning rate to 0.001. The number of parameters in the improved SMLMER-ResNet model of 126,540 is higher than the number of parameters in the SE-ResNet model of 123,780, but recognition accuracy is improved from 88.1% to 99.4%, which we consider acceptable By comparing several commonly used identification algorithms, we efficiently and accurately classified pathological sections of diffuse glioma in compliance with the classification method introduced by the WHO in 2016. Average classification accuracy values were 97.32% and the Kappa coefficient was 0.954. Compared with other deep learning methods, the SMLMER-ResNet module proposed in this paper can effectively extract spatial features and channel features from micro-hyperspectral images, achieving better classification results with less computational cost and increased robustness. Compared with traditional machine learning methods, the model used in this paper can automatically learn relevant features from hyperspectral images and continuously optimize network parameters through backpropagation, which is more suitable for the accurate classification of microscopic hyperspectral images.

## 5. Conclusions

The SMLMER-ResNet algorithm proposed in this paper introduces the channel attention mechanism SE module before the traditional residual connection module. Building on this design, the algorithm introduces a parallel multi-scale image feature method to further extract relevant features from three-dimensional hyperspectral images. Our design improves the convolutional layer within the residual connection by converting it into multi-branch modules with different image sizes, which effectively reduces redundant information and improves the feature extraction ability of the network. This study effectively improves the accuracy of subtype identification in glioma pathology sections compared to conventional algorithms. Using this method, we can reach deep into the atlas information of glioma pathological sections, and identify diffuse glioma pathological sections efficiently and accurately according in compliance with the classification criteria introduced by the WHO in 2016. Our approach solves the problems associated with the poorly efficient, time-consuming, and laborious classification of malignant grades that limit existing auxiliary detection methods. Our study provides an effective tool for auxiliary diagnosis that can be effectively used by clinical pathologists.

However, our work has some limitations. We obtained a small sample size for this study. In the future, we will increase our sample size and make full use of the available clinical resources at the Third Hospital of Jilin University to further validate our model, with the goal of promoting the adoption of our hyperspectral image analyzer within the clinical community. Another of our future endeavors is to explore which particular hyperspectral image bands or pathologic features in hyperspectral images improve the classification results and work with hospitals to complete studies of medical mechanisms and improve the interpretability of the test.

## Figures and Tables

**Table 1:** Comparison of results for different dimensionality reduction methods.

				OA%			AA%	Kappa
		Normal	Grade 1	Grade 2	Grade 3	Grade 4		
Simulated	SVM	83.381	82.186	83.292	83.347	81.911	82.823	0.875
annealing	DT	79.291	76.322	78.676	78.747	77.452	78.097	0.776
algorithm (SAA)	RFR	77.852	74.667	75.781	74.575	73.866	75.348	0.648
Successive	SVM	83.182	83.823	81.456	82.902	81.824	82.637	0.68
Projections	DT	77.442	76.714	77.185	77.943	78.538	77.564	0.702
Algorithm (SPA)	RFR	97.297	78.383	75.267	71.332	68.989	78.253	0.3803

**Table 2:** Comparison among evaluation indicators for different learning rates.

Learning Rate			OA%			AA%
	Normal	Grade 1	Grade 2	Grade 3	Grade 4	
0.1	97.602	94.633	97.792	97.863	95.628	96.704
0.005	97.681	97.587	97.796	96.216	98.982	97.652
0.001	99.705	98.746	97.633	96.992	98.785	97.32
0.0005	99.701	98.874	97.342	96.99	98.689	98.319

**Table 3:** Comparative effectiveness of classification mechanisms.

	Classification Mechanism Settings	FS	FR	FRL2	FSL2
	OA(%)	95.33	94.37	96.64	97.46
Evaluation indicators	AA(%)	91.60	90.59	94.38	96.38
	Kappa(%)	91.24	92.88	95.27	98.28

**Table 4:** Comparison of the effectiveness of attentional mechanisms.

	Settings	Without	Spectral	Spatial	This Study
	OA(%)	90.37	90.89	90.37	97.46
Evaluation indicators	AA(%)	84.59	86.66	86.96	96.38
	Kappa(%)	78.62	82.67	82.48	98.28

**Table 5:** Comparison of model results before and after improvement.

Hierarchical Model			OA/%			AA/%	Kappa	Parameter Quantity
	Normal	Grade 1	Grade 2	Grade 3	Grade 4			
SE-ResNet	89.687	88.663	86.383	84.562	84.353	86.729	0.881	123,780
SMLMER-ResNet	99.705	98.746	97.633	96.992	98.785	97.32	0.954	126,540
SVM	83.381	82.186	83.292	83.347	81.911	83.297	0.875	\
DT	79.291	76.322	78.676	78.747	77.452	77.766	0.833	\
RFR	77.852	74.667	75.781	74.575	73.866	75.926	0.815	\
CNN	82.181	81.112	83.296	82.158	79.132	82.926	0.866	\
2D_CNN	86.589	87.312	86.729	87.132	79.132	86.630	0.889	\
3D_CNN	89.283	88.382	86.256	87.696	89.587	90.862	0.934	\

## Data Availability

Data underlying the results presented in this paper are not publicly available at this time but may be obtained from the authors upon reasonable request.
